# Addressing adolescent substance use with a public health prevention framework: the case for harm reduction

**DOI:** 10.1080/07853890.2022.2104922

**Published:** 2022-07-28

**Authors:** James Michael Winer, Amy M. Yule, Scott E. Hadland, Sarah M. Bagley

**Affiliations:** a Section of General Internal Medicine, Department of Medicine, Boston University School of Medicine, Boston, MA, USA; b Grayken Center for Addiction, Boston Medical Center, Boston, MA, USA; c Department of Psychiatry, Boston University School of Medicine, Boston, MA, USA; d Division of Adolescent and Young Adult Medicine, MassGeneral Hospital for Children, Boston, MA, USA; e Department of Pediatrics, Harvard Medical School, Boston, MA, USA; f Division of General Pediatrics, Department of Pediatrics, Boston University School of Medicine, Boston, MA, USA

**Keywords:** Adolescents, harm reduction, substance use disorder, addiction

## Abstract

Adolescence is a developmental stage defined in part by risk-taking. Risk-taking is critical to normal development and has important benefits including trying new activities and exploring new relationships. Risk-taking is also associated with the initiation of substance use. Because substance use often begins in adolescence, much focus has been on primary prevention with the goal of preventing initial substance use. Secondary or tertiary prevention approaches, such as counselling to eliminate substance use or offering treatment, are common approaches for adolescents with problematic substance use or a substance use disorder. While this is important, for some adolescents, treatment or cessation of use may not be desired. In these cases, Healthcare Practitioners (HCPs) can offer clear advice that incorporates harm reduction. Harm reduction, which is often applied for adults who use substances, reduces the negative impacts associated with drug use without requiring abstinence. Harm reduction is crucial to keeping adolescents safe and healthy and can offer opportunities for future engagement in treatment. The objective of this review is to describe strategies for integrating harm reduction principles in clinical settings that are developmentally appropriate. A patient-centered, harm reduction approach can validate perceived benefits of substance use, offer strategies to minimise harm, and advise reduction of use and abstinence.

**KEY MESSAGES:**
- Substance use often begins in adolescence and traditional approaches are often rooted in prevention framework.
- Harm reduction should be incorporated for adolescents with problematic substance use or a substance use disorder.
- This review offers strategies for integration of harm reduction principles tailored towards adolescents.

## Introduction

### Risk-taking during adolescence

Adolescence is critical developmental period marked by tremendous growth as a youth transitions from childhood to adulthood [1]. Adolescents have the important task of exploring their environments and developing skills that will help them succeed as adults. Risk-taking and experimentation, although sometimes considered negative attributes, are key components of this developmental stage. Through risk-taking and experimentation, adolescents try new activities, discover new academic interests, and explore relationships all of which contribute to independence and autonomy. Adults, including Healthcare Practitioners (HCPs) can play an important role in supporting adolescents’ navigation of risks and promoting practices that minimise harms.

The tendency to try novel experiences can lead some adolescents to engage in behaviours associated with potential harm, such as substance use. Early onset of substance use is associated with development of substance use disorder (SUD) (i.e. nicotine, alcohol, non-medical use of prescription medication, and other substances) later in life [2–4]. Substance use is also associated with short-term risks including motor vehicle crashes, increased prevalence of sexually transmitted infections, and higher rates of non-accidental injury [5,6].

Over the past twenty years, substance use among adolescents has decreased to the lowest levels in decades and more adolescents do not use substances than do use substances [7,8]. These changes should be celebrated and these data can also be shared with adolescents. For some adolescents, knowing that fewer adolescents are using may be empowering. However, it is always critical to validate the adolescent’s experience, which may be that “everyone” in their peer group is using substances. Furthermore, certain substance use, including alcohol and cannabis, continues to be common both among their peers and in the wider community. In 2020, 10%, 20% and 34% of 8th, 10th, and 12th graders respectively reported drinking alcohol in the prior 30 days [9]. In addition, the emergence of vaping has led to an increase in nicotine and cannabis use. Although vaping rates plateaued in 2020, there is still significant concern about the risk for transition to combustible nicotine among adolescents who vape. In addition, 20% of adolescents report ever vaping cannabis, and 1.1% of 8th graders, 4.4% of 10th graders, and 6.9% of 12th graders reported daily cannabis use in 2020. This is the highest recorded level since 1991. Perhaps most importantly, whereas there has been a decline in use of many substances among adolescents, these public health successes have not been shared equally. Significant racial and ethnic disparities exist, with Black and Hispanic adolescents, as well as adolescents from lower socioeconomic backgrounds, demonstrating lower declines in substance use [10]. Additionally, LGBTQ + adolescents are more likely to report recent substance use and problematic use practices than their cisgender heterosexual peers [11,12]. As adolescent substance use epidemiology continues to evolve, the urgent issue of how to talk with and give advice to adolescents who use substances remains, as does the necessity of understanding and addressing these inequities.

### A prevention framework to address adolescent substance use

Many efforts focussed on adolescent substance use focus on primary prevention. Primary prevention involves strategies or interventions that intend to prevent the disease or condition before it occurs [13]. There are many examples of successful primary prevention programs, (Communities that Care, Life Skills Training, Project ALERT, Preventure, and Strengthening Families), and these are in part responsible for the decreased prevalence of SUD among adolescents [14]. For a more in-depth discussion of prevention programs, readers can refer to the recent systematic review by Tremblay et al. in Paediatrics [15].

For adolescents who have begun to use substances, have intermittent use, or who have developed a SUD, secondary and tertiary prevention interventions are necessary. Secondary prevention allows for early identification of high-risk populations resulting in the slowing or stopping of disease progression. Screening, Brief Intervention, and Referral to Treatment (SBIRT) is an effective approach to identify adolescents who use substances and is recommended by the American Academy of Paediatrics (AAP) [16] Tertiary prevention interventions offer treatment and rehabilitation after diagnosis. In cases when an adolescent has problematic substance use or a SUD, there is an opportunity for HCPs to integrate strategies to minimise future harm through harm reduction strategies in addition to advice to stop use or engage in treatment.

### Why harm reduction?

According to Harm Reduction International, harm reduction refers to “policies, programmes, and practices that aim to minimize negative health, social, and legal impacts associated with drug use, drug policies, and drug laws.” [17] In recent times, harm reduction has been synonymous with providing opioid overdose education, overdose reversal agents (e.g. naloxone), and safer injection equipment and spaces for adults who use substances. However, in considering the definition of harm reduction, it is possible to imagine how harm reduction principles can be applied for all kinds of substances and for other age groups, including adolescents. This is especially important as among youth, the rate of overdose deaths that involve opioids plus another substance is higher than opioid alone [18]. Although evidence-based treatment may be effective at reducing or eliminating substance use, for some adolescents, treatment and abstinence may not initially be the goal. Others may not have a use disorder, and instead need support in reducing the potential harms of substance use. For HCPs working for adolescents, determining how to navigate this is challenging.

HCPs caring for adolescents often appropriately focus on preventing, avoiding, or delaying substance use. Often, discussions about substance use are focussed on risks of use. However, prior research has found that adolescents who engage in risky behaviours report knowledge of risks, yet report social factors or perceived benefits are more salient [19]. Focussing on education around risk is an important and valid approach given the known impact that substance use has on brain development and functioning in addition to the association of the adverse consequences of substance use [20]. However, a patient-centered approach can validate perceived benefits of substance use, offer strategies to minimise harm, and advise reduction of use and abstinence. By creating a collaborating relationship with adolescents and affirming their experiences, HCPs will maximise the potential to improve outcomes. For example, for a teen who is having anxiety symptoms and has relief from cannabis, validating the perceived benefits of cannabis use is critical to ensure providing a responsive plan that includes addressing a potential anxiety disorder.

Providing adolescents with tools to reduce health-related consequences to their behaviours is common [21]. For example, education about safer sex to prevent sexually transmitted infections and pregnancy is the standard of care. Pre-exposure prophylaxis (PrEP) is approved for adolescents at risk for HIV [22]. Providing the support to make decisions to mitigate harms is squarely within the scope of HCPs who work with adolescents. A recent qualitative study of youth from different peer groups and communities explored the uptake of informal harm mitigation strategies when using substances in their peer groups. They found that youth experiences, perspectives, and strategies varied and are deeply set within geographical, social, and cultural contexts [23]. Thus, in order to address the inequities faced by adolescents who use drugs, it will be important for HCPs to have an understanding of the context in which their patients live and participate in substance use behaviours.

## Incorporating harm reduction into practice

### Applying harm reduction principles and practices to adolescents

For HCPs, harm reduction can be challenging because there are risks (and maybe fears) of condoning substance use. However, it is possible to both provide clear advice about the potential harm of substance use while also keeping open lines of communication with adolescents by giving guidance about minimising harms. The potential effect on adolescents of feeling that they must hide their substance use or not ask questions because they fear a punitive response is serious and undermines the potential positive impact that HCPs can have. HCPs can take a collaborative approach to working with adolescents by asking about the potential benefits of use. Such information can help the HCP in offering strategies to minimise use. For example, as noted above an adolescent with underlying anxiety may use cannabis to help with symptom control. By the HCP acknowledging that benefit, they can work together to think about other ways to address anxiety symptoms. Practically, this means acknowledging the potential short-term benefits and validating the adolescent’s reality of the positive impact of substance use. Furthermore, not all adolescents who use substances have a diagnosable disorder and SUD treatment may not be indicated. There is, of course, the important caveat of safety considerations. For minors, under the age of 18 years, there is an expectation that adults (caregivers, HCPs, and other key community members such as teachers) will intervene when there is an urgent safety consideration. This remains true when considering substance use.

The goal of this manuscript is to provide brief overviews of some of the most commonly used substances and provide practical advice about how to incorporate harm reduction principles into clinical practice. There are limited data to support some of the following clinical suggestions and it is based on best available evidence and clinical experience. We will briefly also discuss best evidence for treatment options for use disorders. This is not meant to be a comprehensive discussion of all treatment options and instead we have written the following with the assumption that some adolescents will not want to decrease their use, but still should receive advice from HCPs. Many of the same principles and strategies can be applied for multiple kinds of substances and integrated throughout should be a consistent message of compassion and reducing the potential harms of adolescent substance use (Table 1).

**Table 1.** Considerations and strategies for integrating harm reduction principles into adolescent care.

Topic	Considerations	Potential strategies
General approach	Evidence-based prevention and treatment strategies may be effective at reducing or eliminating substance use, however treatment and abstinence may not initially be the goal of the adolescent. In addition, adolescents without a substance use disorder can still be supported in behaviours to minimise potential harms of substance use.	Create multiple and accessible touch points for a non-judgmental and welcoming setting. Offer rewards for behavioural changes related to both substance use or other desired changes. Build trust and recognise that adolescents may not want to pursue abstinence at initial visits. Normalise a positive response to questions about stigmatised behaviour. Accommodate adolescents to allow for easier follow-up: same day visits, tele-health visits, texting for appointments. Do no require abstinence for continued engagement.
Understanding motivations	Adolescents may use substances for a variety of reasons including to address mood symptoms or to connect with peers. Understanding the reasons can help HCPs suggest ways to reduce harms and potentially reduce use.	When asking about patterns of substance use, include questions about potential benefits and motivations for use. If an adolescent cannot answer about potential benefits, ask about the setting where use occurs. For adolescents with co-occurring mental health disorders, facilitate access to treatment.
Leveraging family and social connections	Adolescents are often afraid that disclosure of their substance use will be revealed to their caregivers or have legal consequences, which increases likelihood of withholding information from providers and returning for care.	Assess for friends or other social supports that can reinforce a harm reduction approach. Set-up caregiver only visits when appropriate to allow for navigation of their concerns and questions while maintaining patient confidentiality. Educate families on overdose prevention, benefits of medications for opioid use disorder, naloxone, and other harm reduction strategies. Discuss safe medication storage in a locked location, when possible give responsibility to caregivers to hold and dispense medication.
Decreasing risk of overuse or intoxication	It is important for HCPs to share potential harms associated with substance use and that no use is the safest approach. At the same time, this may leave adolescents with no clear guidance about how to minimise potential risks.	Counsel on pacing alcohol use, alternating alcohol use with water, drinking beverages with a lower alcohol content, and having friends who can be supportive. Discuss timing of use, for example not before driving, going to school, or playing sports (or other activities when sobriety is important). Consider shifting from a cannabis product with high THC content to lower THC concentrate (e.g. vaping hash oil to smoking plant). Educate on withdrawal symptoms and management to help adolescents anticipate symptoms and provide reassurance that HCPs can help treat the symptoms.
Reducing risk of driving related injury	Substance use is also associated with short-term risks including motor vehicle crashes.	Develop protective behavioural strategies to minimise negative effects of substances, this can include plans to get home if in a social setting where there is alcohol or substance use. These can include having a designated driver, agreements with parents or other trusted adults about being able to call without risk of consequences.
Overdose risk reduction	Overdose deaths are increasing despite a decline in adolescent substance use. Additionally, the rate of overdose deaths that involve opioids plus another substance is higher than opioid alone. All adolescents can be taught about overdose prevention to protect themselves and their friends.	Facilitate access to naloxone kits either through distributing kits directly in clinic, community distribution, or pharmacies. Offer medications for opioid use disorder which are the most potent protection from overdose. Educate on possible fentanyl or fentanyl analogue contamination and risk of overdose. Facilitate access to drug checking including Fentanyl Test Strips (pending state legality). Advise about the risks of combining multiple substances and the increased risk of overdose. For adolescents who use alone and are at risk for overdose, provide resources such as the Brave Coop or Canary app which can detect an individual’s inactivity and send emergency response to address potential overdose.
Infectious Disease transmission	While injection drug use is rare in adolescents, they are more likely to engage in behaviours that placed them at risk of overdose and infectious complications like hepatitis C (HCV), hepatitis B (HBV), human immunodeficiency virus (HIV), skin and soft tissue infections (SSTI), and endocarditis.	Ask adolescents how they use substances (intranasal, intravenous, smoking, rectal) and counsel on safe practices. Offer comprehensive HIV, viral hepatitis, and bacterial sexually transmitted infection testing. Vaccinate against hepatitis A and B. Prescribe post-exposure prophylaxis and pre-exposure prophylaxis for HIV prevention. Talk to adolescents about community access to safe injection and smoking supplies. If adolescent injection, ask how they do so and discuss sterile technique and safer drug preparation.
Underlying all of these strategies is the provision of compassionate care that validates adolescents’ experiences and motivations, is non-judgmental, assures them that they have value, and gives gratitude to them for trusting the HCP.		

### Nicotine

Almost 90% of adults whose smoke combustible tobacco products first tried smoking before the age of 18 and 99% by the age of 26 and therefore preventing initiation of smoking remains critical [24]. As the result of successful public health campaigns, use of combustible tobacco products has significantly declined among adolescents over the last couple of decades. In 2020, 4.6% high school students reported past 30 days cigarette use compared to 15.8% in 2011 [25]. For adolescents who have not ever smoked or recently smoked, in addition to providing positive feedback about their decisions, HCPs can explore with adolescents the reasons for not smoking and help validate their choices.

For adolescents using nicotine who are not ready or motivated to quit, there are other options for HCPs to reduce the harms of ongoing use. They can assess reasons for use in addition to readiness to quit, and by identifying underlying reasons, offer more tailored advice about how to cut back. For example, for adolescents who are smoking because it provides a relief from mood symptoms, a HCP may focus on engaging them with behavioural health treatment. HCPs can offer “abstinence” challenges, or as commonly referred to by adolescents, “a tolerance break”, to evaluate to see if they notice any changes in concentration, motivation, or other symptoms they may find functionally inhibiting [26]. One strategy is to help adolescents plan short periods of time or limiting the number of cigarettes smoked per day. These can be especially helpful when an adolescent feels they can easily stop at any time. Additionally, when introducing nicotine replacement therapy (NRT), HCPs can suggest alternating days of NRT use. To help prepare adolescents for when they stop nicotine use, HCPs should review signs and symptoms of nicotine withdrawal so they are not surprised if they experience increased anxiety, insomnia, or headaches. Adult data suggests that an individual may need multiple quit attempts prior to successfully quitting; one recent study found on average adults have 30 attempts before having long-term success [27]. HCPs should be prepared to have multiple touch points with adolescents before any changes in behaviour may occur. HCPs can work with adolescents and caregivers to determine positive rewards for changes in behaviour that are associated with cutting back and offer NRT to alleviate withdrawal symptoms.

Adolescents with a nicotine use disorder, who are younger, and have co-occurring mental health conditions are less likely to quit [28]. Behavioural treatment for nicotine use disorder, including cognitive behavioural therapy and motivational enhancement therapy have the strongest evidence to assist in cessation attempts [29]. Contingency management, providing positive rewards for changes in behaviours shows some promise for smoking cessation in adolescents including in web-based remote forms [30–32]. There are no US Food and Drug Administration (FDA)-approved pharmacologic treatments for adolescents under the age of 18 years with nicotine addiction. Additionally, a recent systematic review for adolescents up to age 18 found that neither bupropion SR nor NRT demonstrated an effect on smoking cessation in adolescents [33]. The website smokefree.gov, contains an adolescent section that has helpful resources including an app, QuitSMART, that provides regular positive reinforcement for adolescents to quit smoking and has flexibility for adolescents who are considering but not yet ready to quit [34].

### Cannabis

Although there has been an overall decline in adolescent substance use, cannabis use has remained fairly constant over the past ten years. In 2020 past 30-day cannabis use was 6.5%, 16.6%, and 21.1% among 8th, 10th, and 12th graders respectively, with 6.9% of 12th graders using cannabis daily [9]. Cannabis can be consumed in a variety of ways including smoking the plant, eating or drinking food or drinks with cannabis (“edibles”), and vaporising concentrates. These cannabis products have varying levels of the psychoactive component of cannabis, delta-9-tetrahydrocannabinol (THC). Notably, the THC content in cannabis plants has increased substantially since the late 1990s from 4% in 1995 to 14% in 2019 [35]. Furthermore, concentrates such as wax or hash oil have very high THC levels ranging from 39% to 69% [36]. Adolescents are often able to use cannabis edibles and concentrates with less risk for detection since both products are not associated with the smell of smoking the plant and can be easily concealed.

It is important to determine the type of cannabis product that an adolescent is using since the acute and short-term adverse effects associated with cannabis are largely related to THC content. Specifically, high levels of THC are associated with anxiety, agitation, paranoia, and hallucinations [36]. Higher potency cannabis products have also been associated with an increased risk for the development of a cannabis use disorder [37]. A harm reduction strategy for cannabis includes encouraging adolescents to shift from the use of cannabis concentrates to the cannabis plant. Adolescents may also view cannabis products from a dispensary as safer than products obtained from a different source. It is important for adolescents to be aware that quality control and oversight of dispensaries is variable since cannabis is still categorised as a schedule I substance. Furthermore, edibles from dispensaries are not routinely labelled with a serving size, and adolescents have experienced acute mental status changes after inadvertently consuming large doses of THC from an edible [38].

HCPs may encounter many adolescents who are motivated to briefly stop cannabis use because they have developed a tolerance to cannabis and are having to use a greater quantity to have the same effect. In these cases, HCPs can work with the adolescents who want to take a tolerance break [26]. If an adolescent who has been using cannabis regularly decides to stop their cannabis use for a period of time, it can be important to educate them on the signs and symptoms of cannabis withdrawal. Cannabis withdrawal symptoms can begin within a day of cessation, peak at approximately a week, and last for up to two weeks [39]. The symptoms include aggression, anger, anxiety, irritability, insomnia, decreased appetite, and stomach pain [40]. Although there are no established treatments for cannabis withdrawal it is important for adolescents to understand their experience since withdrawal symptoms may be a barrier to cessation of use.

#### Considerations for the use of electronic vaping devices for nicotine and cannabis use

In 2018 the Surgeon General issued an advisory on electronic cigarette (e-cigarette) use among youth in response to significant increased use [41]. E-cigarettes are devices that can vaporise a nicotine solution combined with liquid flavours which have become more popular and socially acceptable amongst adolescents [42]. Since 2014, they have been the most commonly used tobacco product among adolescents [43]. In addition to the concerns for the harms caused by e-cigarette aerosols including the concerning condition of electronic-cigarette/vaping associated lung injury (EVALI), an association between e-cig use and a transition to combustible tobacco has also been described [44–46]. In addition, adolescents can also vape cannabis hash oil and this is becoming more common. Vaping cannabis more than doubled among adolescents from to 2013 to 2020 and past 30-day use increased seven fold from 2013 to 2020 (1.6–8.4%) [47]. This may reflect both a preference for vaping as well as evidence for the concern that vaping provides a higher potency and may lead to development of a cannabis use disorder more quickly. According to the National Youth Tobacco Survey, in 2021, 43.6% of high school students vaped at least 20 days a month and 27.6% reported vaping each day [48].

For adolescents vaping either nicotine or cannabis, similar approaches as outlined above can be offered in terms of exploring the reasons and motivations for continued use and offer clear advice that the safest plan is to cut back and abstain. However, some adolescents will not be interested in quitting. In other cases, they may have previously smoked cigarettes but transitioned to vaping to decrease their use of combustible products for nicotine delivery. As with any kind of substance use, offering rewards for behavioural change or decreases in use (without cessation) may be a first step as well as identifying the underlying reasons for use. For adolescents using nicotine, HCPs can advise reducing the concentration of nicotine in the cartridges they are using. For those using cannabis, trying to limit or space out the time may be helpful. In addition, HCPs should provide clear advice that driving after vaping cannabis is not safe and to develop plans with their families for safe transport.

### Alcohol

Although alcohol use among adolescents has generally decreased over time, in 2021 past 30-day alcohol use was 7%, 13%, and 26% among 8th, 10th, and 12th graders respectively [7]. Young adults continue to have the highest prevalence of alcohol use, including binge alcohol use [49]. Binge drinking typically begins in adolescence and is defined differently than adults, as adolescents are more likely to have higher blood alcohol concentrations after drinking similar amounts of alcohol. Binge drinking in adolescents is defined as 3 or more drinks for 9–13 year olds, as 4 or more drinks for boys and 3 or more drinks for girls aged 14 or 15, and as 5 or more drinks for boys and 3 or more drinks for girls aged 16 or 17 [50]. Compared with adults, adolescents tend to consume higher quantities of alcohol per occasion but drink less frequently [51].

Treatment of adolescents with alcohol use disorder is similar to treatment of adults with a combination of behavioural treatment and pharmacologic treatment, although there are not currently FDA approved medications for alcohol use disorder for under 18 years. However, some adolescents may not meet criteria for an AUD and others may want to continue to drink alcohol. For those adolescents, it is important to share clear guidance about the potential harms associated with alcohol use and that no use is the safest approach. The acute effects of alcohol use are associated with immediate harms such as blackouts, increased risk of motor vehicle accidents or making decisions about sexual behaviour or risk taking that place them at harm for physical or sexual consequences. However, there are additional steps that HCPs can take to reduce risk for adolescents who use alcohol. For example, HCPs can help adolescents develop protective behavioural strategies (PBS) for how to reduce the negative consequences from drinking [52]. For example, advising adolescents on how to get home if in a social setting where there is alcohol. In those cases, if possible, engaging with caregivers to assure the adolescent that calling for a ride is a safe approach and they will not be punished is important. Other strategies such as planning to try to drink less than usual, pacing alcohol use, alternating alcohol use with water, drinking beverages with a lower alcohol content, and having friends who can be supportive are possibilities. Finally, validating the potential benefits of alcohol use and addressing underlying reasons why the adolescent is drinking should be part of the discussion.

### Opioids

The overdose epidemic has not spared adolescents and overdose related deaths rose three-fold from 2019 to 2021. Similar to adults, these deaths are increasingly driven by synthetic opioids and most often involve other kinds of substances. Further inequity among adolescents experiencing overdose deaths is highlighted by these recent findings, showing that American Indian and Alaska Native adolescents experienced the highest overdose rate, followed by Latinx adolescents [53]. The same evidence-based strategies to reduce risk of opioid-related overdose, treatment with medications for opioid use disorder and distribution of naloxone, that are offered to adults should also be offered to adolescents [54,55]. Employing harm reduction practices for adolescents who use opioids or with opioid use disorder is important to reduce morbidity and mortality and should be tailored to their current risk profile [56].

#### Overdose prevention

Adolescents are generally uninformed about overdose risk [57]. When working with adolescents and their families, overdose education should always be provided. Offering education and distribution of naloxone to families of people who use substances, and their community support networks has been shown to increase uptake of naloxone and reduce risk of overdose death [58,59]. All 50 states, the District of Columbia, and Puerto Rico allow an individual to obtain naloxone without a prescription in some capacity and HCPs should be aware of their state specific access in order to counsel adolescents [7]. Additionally, the majority of states have also enacted laws which protect laypeople who administer naloxone in an emergency from civil and/or criminal liability [60]. HCPs should be aware of their state Good Samaritan Laws in order to empower adolescents to use naloxone and call for help when responding to an overdose [60].

HCPs can also recommend adolescents reduce risk by using with other people around, tester shots, and using fentanyl test strips (FTS) [23,61–63]. Since fentanyl and fentanyl analogues (FFA) are the most common substance found in opioid overdose deaths, FTS are a harm reduction tool to detect possible fentanyl that have been shown to be acceptable amongst young adults [64,65]. HCPs can teach adolescents how to test a sample of drug residue or crushed whole pressed pill with the test strip as pills may only contain fentanyl in a certain area. In addition, they can advise patients that current market available strips may not detect all high potency synthetic opioids (e.g. carfentanil) that are present in the drug supply and that dilute samples may lead to false-negative tests [66]. Therefore it is important to use this tool in conjunction with other additional harm reduction practices mentioned (i.e. using with others, carrying naloxone). However, it is important for HCPs to know the laws in the areas where the practice. A recent systematic legal analysis by Davis et al. [67] found that it is only clearly legal to possess drug checking equipment like FTS in 22 states with some states only allowing them to be obtained from syringe service programs [68].

For some families, introducing the idea of being trained in overdose prevention, including naloxone administration, may be scary and overwhelming. Similar to other sensitive topics that HCPs provide guidance about, we suggest using normalising language such as, “I like to talk to all families about how to recognize and respond to an overdose. I hope that you will never need to use this information, but want to make sure that you are prepared just in case.” After that introduction, the HCP can assess the patients and families’ comfort with overdose prevention and provide naloxone.

#### Medications for opioid use disorder

The most effective approach to reduce the risk of overdose is to offer medications for opioid use disorder (MOUD) [54,69]. In adults, treatment with buprenorphine and methadone is associated with reduced all-cause and opioid-related mortality [68,69]. All adolescents should be offered MOUD if diagnosed with moderate to severe opioid use disorder [55,70–72]. The current FDA approved MOUD include methadone, buprenorphine, and naltrexone. Only buprenorphine is approved for 16 years and older; methadone and naltrexone are both approved for 18 years and older but in some cases may be used off-label for younger ages. For a complete review of MOUD for adolescents, HCPs can reference the recent manuscript in Paediatrics by Robinson and Wilson [56].

There are no definitive guidelines for how long to continue MOUD for adolescents with opioid use disorder. Some studies have shown that relapse and problematic use decrease the longer adolescents are on MOUD [73,74]. Thus, for adolescents with severe opioid use disorder who are stabilised on MOUD, HCPs should consider longer treatment and ongoing discussions with the patient about the potential risks of discontinuation [75].

#### Additional harm reduction supplies and teaching

Most syringe service programs do not provide services to individuals under the age of 18 years, although injection drug use is rare in this age group [7]. However, adolescents who inject tend to have riskier use patterns than older adults including reuse of syringes, sharing of syringes and equipment, and lower likelihood of having been tested for infectious complications. They are more likely to engage in behaviours that placed them at risk of overdose and infectious complications like hepatitis C (HCV), hepatitis B (HBV), human immunodeficiency virus (HIV), skin and soft tissue infections (SSTI), and endocarditis [76–79].

HCPs can familiarise themselves with how to counsel on safer injection practices and supplies to help reduce risk. Although focussed on adults, HCPs can reference existing harm reduction guides that includes safer supplies, conditions, and practices to help reduce injection related morbidity and mortality including encouraging use of new sterile syringes and to use one syringe each use [64,80]. Being knowledgeable about supplies and practices is important for HCPs, as adolescents have less access and comfort with traditional harm reduction services including needle exchanges [81]. It should be noted that state and local laws vary on rules for providing harm reduction supplies, especially for minors, and HCPs should know the laws in the areas they work [82,83]. Naloxone, however, is a prescription medication and can be prescribed for minors.

### Benzodiazepines

Benzodiazepines are commonly prescribed for their anxiolytic, hypnotic, muscle relaxant, anticonvulsant, and amnesic properties to aid with anxiety disorders, mood disorders, and sleep problems [84]. In adults, they are indicated for short-term use (2–4 weeks), but their safety has not been established beyond that period and are not approved for paediatric use outside of epilepsy and seizure disorders [85]. Bueshnell et al. examined prescription benzodiazepine use in privately insured children and adolescents in the US and found that anxiety disorders were the most common indication for prescription in adolescents compared to epilepsy as the most common indication in children [84]. The majority of younger age groups reports receiving benzodiazepines from a friend or relative and approximately 1 in 10 US high school seniors report prior benzodiazepine exposure [86,87].

In 2020, the Food and Drug Administration required an updated boxed warning in September 2020 for the “potential for abuse, addiction, and other serious risks” of benzodiazepines [88]. While there is little known about benzodiazepine use and misuse in adolescents <18 years of age, younger adults 18–25 years of age have been found to have the highest increase in rates of misuse [86]. Individuals who initiated nonmedical use of benzodiazepines at 18 years of age or younger in the U.S. were significantly more likely to develop substance use disorders than those who initiated nonmedical use later in life [87]. Furthermore, it is has been found that co-occurring benzodiazepine use has been linked to opioid overdose, and that the rate of overdose deaths involving benzodiazepines has been increasing and adolescents are unaware of overdose risk [89–91].

In 2021, the United States Drug Enforcement Administration issued a Public Safety Alert after a sharp rise in counterfeit (“pressed”) pills made to look like real prescription pills including alprazolam that contain lethal doses of fentanyl [92]. These counterfeit pills are increasingly available in the drug supply and have been found to be obtained online including social media platforms and e-commerce websites [92]. HCPs should educate adolescents and their caregivers on these dangers and provide naloxone to all adolescents who use benzodiazepines, prescribed or non-prescribed, in addition to use of opioids or risk of opioid exposure [93]. HCPs should counsel caregivers of adolescents to be aware of these common sources and to keep medications in a secure location like a lock box. Additionally, fentanyl test strips (discussed in section on OUD) may be another tool to check for the presence of FFA in pressed pills.

#### Discontinuing safely

Extra caution should be used when continuing benzodiazepines in those who meet DSM-5 criteria for sedative-hypnotic use disorder, especially in those with concomitant opioid or alcohol use, given the increased misuse potential and overdose risk [93]. Adolescents with prolonged use of benzodiazepines or benzodiazepine misuse may require a controlled taper to help with withdrawal management and to mitigate risk of seizures [93]. While there is no consensus on preferred taper schedules for adults, existing taper plans for adults for inpatient and outpatient settings can serve as a guide for HCPs who take care of adolescents [93–95]. Additionally, Cognitive Behavioural Therapy has shown benefit for benzodiazepine dependence and sedative hypnotic use disorder when added to a taper schedule [96]. Many adolescents may be using benzodiazepines to treat a co-occurring anxiety disorder and should be offered other evidence-based treatment, such as a selective serotonin reuptake inhibitor, which has less potential for misuse.

### Stimulants

Stimulants include cocaine, methamphetamine, and prescription drugs. Prescription stimulant misuse is the most common, and in 2021 past 30-day use was 4%, 4%, and 3% among 8th, 10th, and 12th graders [7]. While overall stimulant use in adolescents remains low in the USA, stimulant-involved overdoses in adolescents have significantly increased in recent years [97]. Approximately 1 in 25 high school students has used cocaine, and 1 in 50 has used methamphetamine [98]. Roehler et al. found that suspected stimulant overdoses in youth increased quarterly from April 2016 and September 2019, 3.3% for 0–10 year-olds, 4.0% for 11–14 year-olds, and 2.3% for 15–24 year-olds. Nationally, deaths involving cocaine and other stimulants increased sharply after 2016, including among adolescents and young adults ages 15–24 [99,100]. Additionally, stimulant involved opioid overdose deaths have significantly increased and accounted for the majority of polysubstance involved opioid overdose deaths in youth [18]. In adolescents, stimulant misuse is more common with non-medical use of prescription stimulants [101]. The National Survey on Drug Use and Health (NSDUH) estimated in 2019 that 1.5% of adolescents aged 12–17 misused prescription stimulants [49]. Austic et al. found that the peak risk of initiation among adolescents who misuse stimulants to be between age 16 and 19 [102].

Patients who use stimulants should be counselled on the risk of unintentional opioid exposure, and should consider the use of FTS in this setting to decrease the risk of an opioid overdose and be given naloxone [103]. HCPs should also caution on the common practice of using opioids to “come down” after using stimulants and counsel on overdose prevention and not using alone as discussed in the previous section on opioid use. HCPs should ask adolescents how they use stimulants to individualise counselling. For example, adolescents who smoke methamphetamine or cocaine should be instructed to not share their equipment as this practice can transmit HCV or HIV [64].

Unlike opioids, medication options for treatment stimulant misuse and stimulant use disorders continue to be limited and ineffective [104,105]. Current evidence supports contingency management as the behavioural therapy with the most evidence to encourage sustained abstinence from cocaine and methamphetamine use. Adolescent tailored contingency management has been successfully adapted further by using a fish bowl method that provided increasing numbers of draws for prizes from the fish bowl with consecutive samples documenting stimulant abstinence and has been shown to be effective [106].

### Importance of confidentiality and including caregivers

At the beginning of a clinical encounter, HCPs should educate both adolescents and their caregivers about the importance of confidentiality, including the limits of confidentiality if there are acute safety concerns. Confidentiality is a key component of adolescent healthcare and increased adolescent participation and connection with HCPs, and has been shown to increase adolescent participation and connection with healthcare providers [107]. Adolescents are often afraid that disclosure of their substance use will be revealed to their caregivers or have legal consequences, which increases likelihood of withholding information from providers and returning for care [107]. Allowing for a safe space and encouraging adolescents to navigate their health care decisions is important for their cognitive development and in creating a therapeutic relationship with providers [108,109].

In cases when including caregivers is possible, HCPs can help introduce harm reduction strategies to mitigate risk. Regardless of the kind of substance, caregivers can help reinforce minimising the risks of use. For example, an AAP Policy Statement notes because caregivers underestimate the extent of their teenagers’ drinking behaviours, their inclusion in discussions about minimising the risk of drinking can include safer practices like calling their caregivers if they are concerned about friends or themselves driving after using alcohol. Offering to be a meditator in these conversations is an important role for HCPs, and can alleviate some of the worry that an adolescent may have about having them along. They also provide an opportunity to highlight the adolescent’s strength of optimising safety [109]. As Ryan notes, there are “no hard and fast rules as to when parents should be included in discussions about their adolescent’s alcohol use…and is generally a judgement call by the primary medical providers” given that there are no acute safety concerns [110].

Including caregivers of adolescents when possible, can be a powerful tool when integrating harm reduction principles into clinical practice, including discussions around treatment. Consent to treatment is separate from confidentiality, and laws for consent for treatment of minors vary by state and it is important for HCPs to be aware of the laws where they practice [111,112].

## Conclusions

When applying harm reduction principles to adolescent care, there is no one size fits all approach, rather it varies based on communities and peer groups. As we continue to witness the disparate effects of the war on drugs, especially how it has impacted Black and Brown people who use drugs through harsher criminalisation stemming from racist drug laws and policies, it is imperative for HCPs to include adolescents in the development of tailored approaches while always emphasising safety. The goal of harm reduction is preventing and reducing harm, but failure to consider the context in which an adolescent engages in substance use may lead to more harm being done.

Principles of harm reduction that include compassion, understanding, and non-coercion can be applied to working with adolescents who use substances. The important differences and impact of substance use on adolescents compared to adults should be considered in approaches to giving advice and offering treatment to adolescents who use substances. Substances impact brain development and early use is associated with developing an addiction, therefore primary prevention with the goal of abstinence and delaying of use will continue to be of critical importance. Consistent messaging for adolescents that includes feedback that most other adolescents do not in fact use substances can be reassuring for both adolescents and their caregivers. However, for adolescents who use substances, HCPs must be ready to give advice to decrease the associated harms and validate the reasons for adolescents.

## Data Availability

Data sharing is not applicable to this article as no new data were created or analysed in this study.
